# Development of 116 kDa Fraction for Detecting Experimental *Toxoplasma gondii* Infections in Mice

**Published:** 2013

**Authors:** Mohey Abdel-Hafez HASSANAIN, Eman Hussien ABDEL-RAHMAN, Nagwa Ibrahim TOALEB, Raafat Mohamed SHAAPAN, Hasan Ali ELFADALY, Nawal Abdel-Hafez HASSANAIN

**Affiliations:** 1 Department of Zoonosis, Veterinary Research Division, National Research Center, Dokki, Giza, Egypt; 2 Department of Parasitology and Animal Diseases, Veterinary Research Division, National Research Center, Dokki, Giza, Egypt

**Keywords:** *Toxoplasma gondii*, Strains, Mice, Partially purified fraction, ELISA

## Abstract

**Background:**

Serological diagnosis of *Toxoplasma gondii* infection using crude antigens may not be more accurate. To increase the diagnostic potency of antigens, isolation of their immunogenic fractions could be useful. The current research adopted to obtain an affinity isolated fraction from RH strain using CNBr Sepharose 4B column coupled with infected mice sera helping in detection of IgM and IgG of toxoplasmosis due to RH strain and other strains.

**Methods:**

The isolated fraction was characterized by SDS-PAGE. Moreover, the diagnostic potency of the fraction was assessed by indirect ELISA in mice experimentally infected with RH strain and two other local strains; one of sheep origin and the other of human origin.

**Results:**

The fraction was found to be consisted of a single band of 116 kDa compared with 17 bands ranged from 116 to 16 kDa associated with crude extract. The fraction proved potent diagnostic potentials of acute and chronic mice toxoplasmosis. Where it was detected both IgM and IgG antibodies as early as two days and as late as 2 months post experimental infection with any of the three strains. The level of detected IgM and IgG by RH fraction was higher in mice infected with RH strain than with local strains except IgM due to sheep strain parasite.

**Conclusions:**

The 116 kDa fraction of *T. gondii* tachyzoites can be considered as a candidate in improving of serodiagnosisof *Toxoplasma* infections.

## Introduction

Toxoplasmosis is a zoonotic disease caused by the obligatory protozoan *Toxoplasma gondii* being responsible for major economic losses in most classes of livestock through abortions, still birth and neonatal losses (1, 2). Humans can also become infected when they eat undercooked meat with tissue cysts, consume contaminated food or drink and accidentally ingest oocysts from the environment (3). Although most infections in humans are asymptomatic, this parasite can sometimes cause a devastating disease (4).

Toxoplasmosis diagnosis depends on direct and indirect methods. Besides the Sabin-Feldman dye test, which is accepted to be the reference test, serologic tests such as ELISA are means of indirect diagnosis. As detected antibodies in serologic tests are correlated with antigens that cause their synthesis, it is important to know new and different antigens (5). The previous serological studies involved the use of crude antigens in the detection of *T. gondii* antibodies (6). To increase the diagnostic potency of antigens, isolation of their immuno-genic fractions could be useful (7). Tachyzoites stage is thought to be responsible for acute infection and expresses imm-unodominant antigens that induce strong immune responses (8).

Partial purification of tachyzoites antigen was conducted by affinity column chromatography and the purified fraction proved potency in detecting IgG antibody level in immunized mice by ELISA (9). A fraction of 30–33 kDa isolated from crude tachyzoites antigen proved successful in the diagnosis of human toxoplasmosis by ELISA (10). Two proteins in the 20–40 kDa range induced a significant humoral response as revealed by immunoblot assay (11). Abdel-Rahman et al., (12) used *T. gondii* crude and affinity purified tachyzoites antigens isolated from slaughtered sheep in the diagnosis of toxoplasmosis in horses. Conde de Felipe et al., (13) proved that *T. gondii* fractions 29-35 kDa detected a specific peak of IgG in goats two weeks earlier than crude extract. Ghazy et al. (5) introduced affinity purified fractions (bound and unbound) obtained from the locally isolated tachyzoites (equine origin), which were utilized for the first time for detection of *T. gondii* antibodies in horses. They added that bound fraction in indirect ELISA proved better diagnostic potency than both indirect fluorescent antibody test (IFAT) and modified agglutination test (MAT).

Therefore, the objective of the current study was to develop affinity partially purified fraction of RH tachyzoites antigen to be used for serological diagnosis of experimental toxoplasmosis in mice infected with different strains of the parasite.

## Materials and Methods

### T. gondii RH strain

Virulent RH strain of *Toxoplasma gondii* was obtained from colony maintained in Department of Zoonosis, National Research Center by serial passage in mice according to the method of Johnson et al. (14).

### Local T.gondii sheep strain

The strain had been isolated from pooled meat, heart, diaphragm, liver, and esophagus samples that prepared as described by Shaapan and Ghazy (15). Virulent local strain of *T. gondii* obtained by bio-assay of pooled samples in kittens according to the method of Davis and Dubey (16) and maintained in our laboratory by serial passage in mice.

### Local T. gondii human strain

Local human *T. gondii* strain had been isolated from tissue samples obtained from placenta and umbilical cord of aborted fetus from a governmental hospital, digested and bio-assayed in mice according to Sharma and Dubey (17).

### Antigens Preparation

Soluble crude antigens were prepared from tachyzoites of RH strain, sheep and human isolates of *T. gondii*, using the method described by Waltman et al., (18); briefly, tachyzoites5 x 10^6^ per ml were repeatedly freeze and thawed to rupture the parasite wall, sonicated and centrifuged at 12,000 rpm for 45 min at 4^o^C. The supernatant was collected and its protein content was determined by the method of Lowry et al. (19). It was considered as crude antigen and stored at -20°C until use.

### Experimental infection of mice

Three groups each of forty Swiss albino mice with average weight 25-30gm were separately infected with 2x10^6^ RH, sheep and human *T. gondii* strains tachyzoites according method described by Dubey and Beattie (20). The experiment was extended for three months to cover the acute and chronic course of toxoplasmosis. Blood samples were taken from mice at 2, 4, 6, 8, 10, 14, 21, 30, 60 and 90 days post infection. Serum samples were obtained and stored at -20 ^0^C until use.

### Immune-affinity purification of RH antigen

Affinity purification of crude antigen of RH strain was performed as described by Ahn et al., (9) with slight modifications. In brief, *T. gondii* positive sera were obtained from experimentally infected mice. The positive sera were dialyzed against 0.1 M NaHCO3 containing 0.5 M NaCl and 0.02% NaN3 and coupled to Cyanogen bromide-Sepharose 4B (CNBr-Sepharose 4B) swollen beads (Sigma-Aldrich, USA) by strictly using the following the manufacturer instructions. Bound fraction was eluted with 50 mM glycine, pH 2.3 containing 500 mM NaCl.

### Sodium dodecyl sulphate polyacrylamide gel electrophoresis (SDS-PAGE)

Proteins of crude antigen of RH strain and isolated fraction were separately electrophoresed on 12.5% SDS-PAGE according to the method of Laemmli (21). After separation, the gel was fixed in 50% methanol and stained with silver stain according to the method of Wray et al., (22). Molecular weight standards were electrophoresed on the same gel to calculate the relative molecular weights of the examined antigens.

### Preparation of hyperimmune serum

Forty µg of crude RH *T. gondii* antigen per kg of rabbit was mixed with an equal volume of Freund's complete adjuvant and injected subcutaneously into each of 5 rabbits. A booster dose of the antigen in Freund's incomplete adjuvant was injected 14 days later. Second and third booster doses were given on days 21 and 28, respectively (23). Blood samples were collected 4 days post last injection from rabbit's ear vein. Collected antisera were aliquoted and stored at -20 °C until use.

### Enzyme linked immune-sorbent assay (ELISA)

The Diagnostic potency of the fraction was first evaluated against hyper-immune serum raised in rabbits by ELISA. The fraction was also utilized in the detection of IgM and IgG levels in the sera of mice infected with RH, local sheep and human *T. gondii* isolates at 2, 4, 6, 8, 10, 14, 21, 30, 60 and 90 days post infection. The optimum antigen, serum and conjugate concentrations were determined by checkerboard titration and test procedures (24). The cut off values of optical densities (OD) were calculated according to Hillyer et al. (25). Each serum samples was tested in triplicate.

## Results

### Electrophoretic profile

**Fig. 1:**
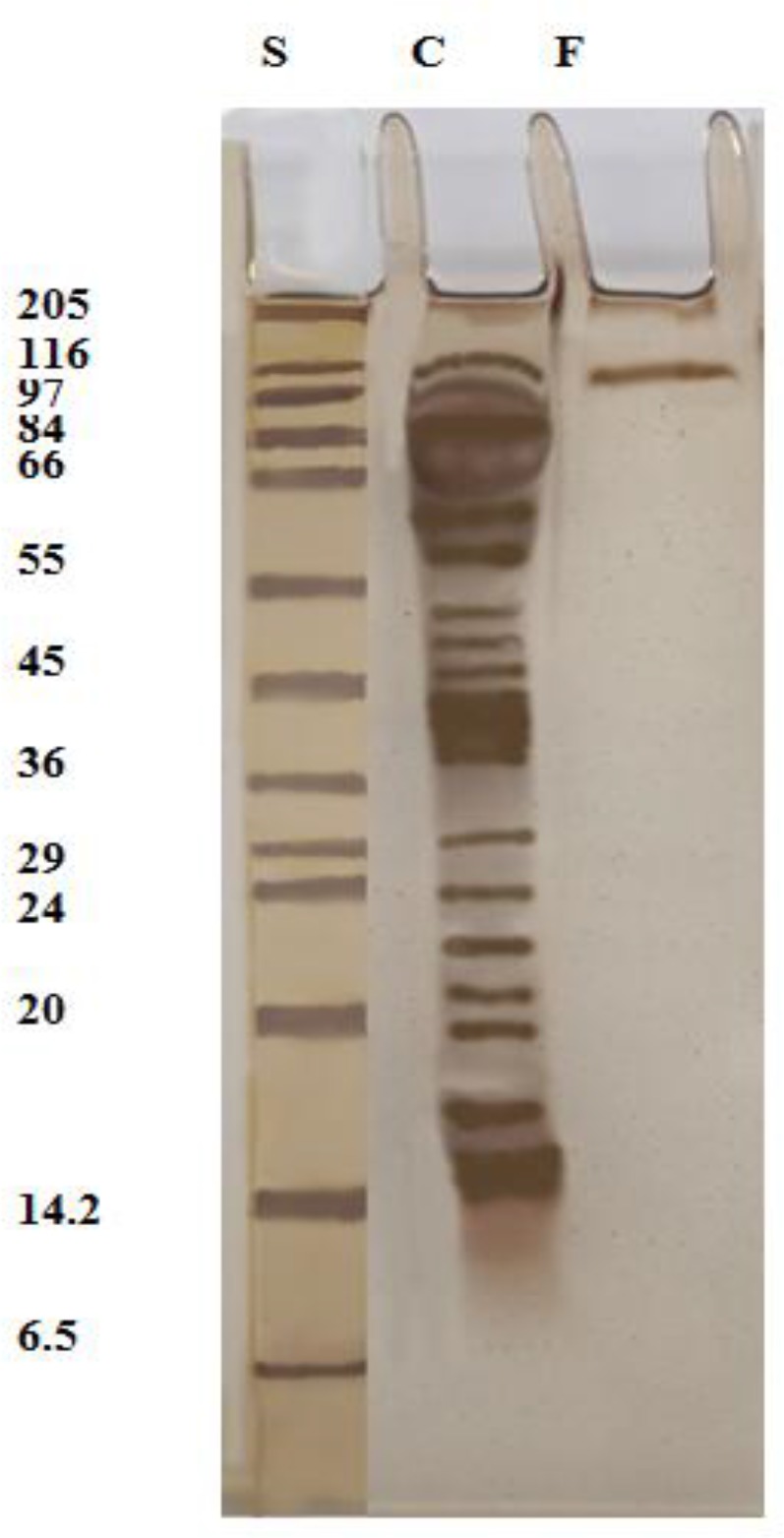
The electrophoretic profile of the isolated fraction (lane F) compared with crude extract of T. *gondii* RH strain (lane C). Molecular weight standards in kDa (Lane S)

The purification process resulted in a fraction identified by experimentally infected sera of mice collected at different intervals of infection coupled with CNBr-Sepharose 4B. The fraction was characterized by SDS-PAGE 12.5% slab gel which resolved into a single band of 116 kDa compared with 17 bands associated with crude extract (116- 98- 90- 64- 60- 58-52- 50- 45- 38- 30- 24- 23- 21- 19- 17 and 16 kDa) (Fig. 1).

### Immunodiagnostic potentials of the fraction

Based on checker board titration results for ELISA the optimum concentration of antigen was 20µg/ml, while the dilution of antibodies was 1: 100 and the dilution of conjugate was 1: 1000. The diagnostic potentials of the fraction were first evaluated in rabbits and then in mice using ELISA. The cut off value was 0.65.

### (A) In Rabbits

**Fig. 2:**
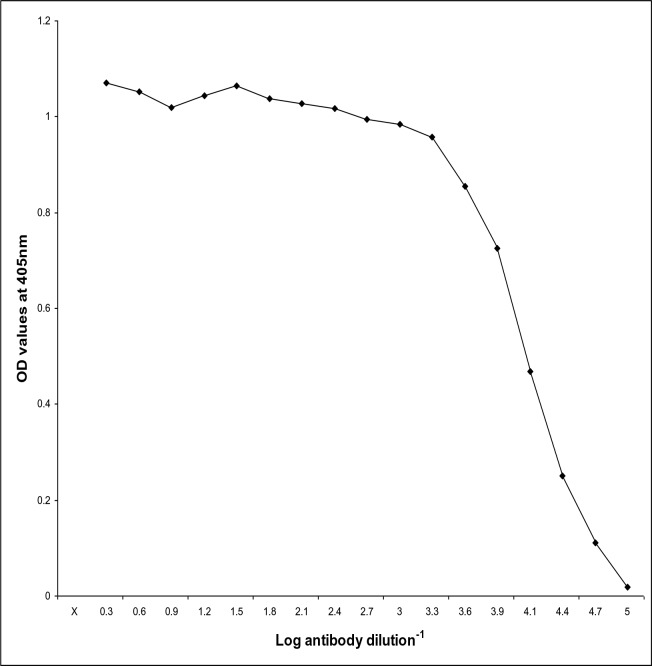
Diagnostic potentials of the fraction 116 kDa of *T. gondii* tachyzoites against hyperimmune sera in rabbits

The fraction proved to have high diagnostic potency against pooled hyper-immune sera raised in rabbits where it detected IgG antibodies in diluted serum reached to 1:128000 (Fig. 2).

### (B) In mice infected with T. gondii RH strain

**Fig. 3:**
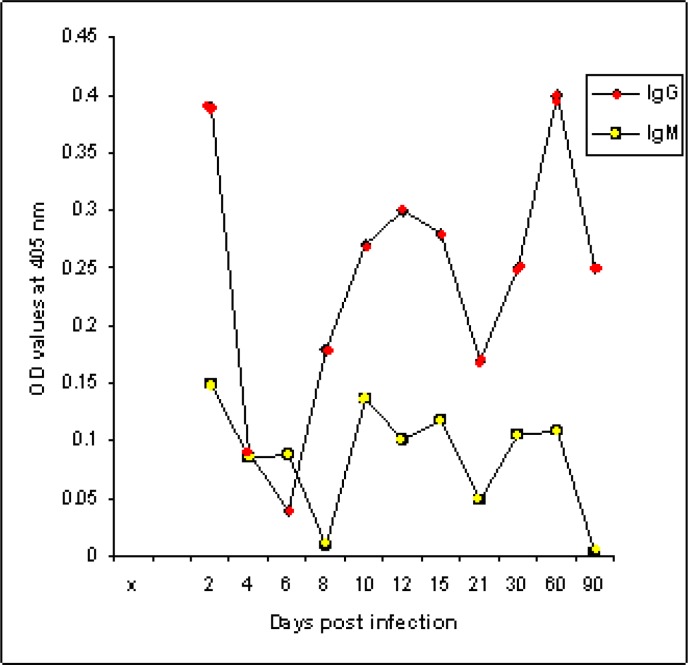
IgM and IgG antibodies levels to fraction 116 kDa of *T. gondii* RH strain in sera of mice experimentally infected with RH strain of *Toxoplasma gondii*

The fraction also proved potency in the detection of IgM level in infected mice sera at early stage of infection; 2 days and the OD value was 0.15. Unexpectedly, the fraction also detected IgM response at late stage of infection; 30 and 60 days recording OD values 0.106 and 0.109 respectively as shown in fig. 3.The fraction was successfully utilized in the detection of IgG level in the sera of mice experimentally infected with *T. gondii* particularly at early stage of infection (2 days) recording OD value reached to 0.388 and at late stage of infection (2 months) with OD values 0.402 (fig. 3).

### (C) In mice infected with T. gondii human isolate

**Fig. 4:**
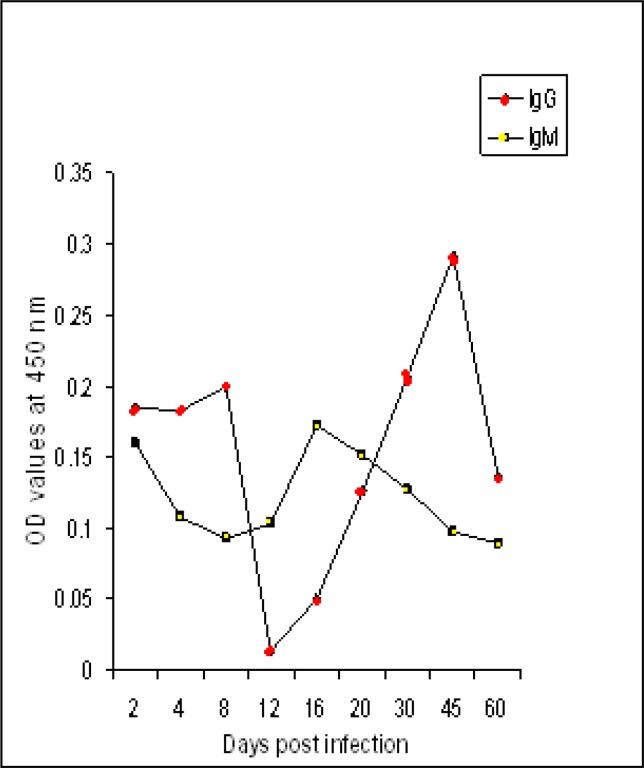
IgM and IgG antibodies levels to fraction 116 kDa of *T. gondii* RH strain in sera of mice experimentally infected with human isolate of *Toxoplasma gondii*

The level of IgM was low at early stage (2days) of infection (0.16). Then the level was fluctuated between increase and decrease until the end of the experiment. The level of IgG started low (0.185) but higher than IgM then it began to increase starting 30 days post infection and reached its peak after 45 days after infection and then decreased. The fluctuation of antibodies levels is intervals dependent belong to killed mice in interval times (Fig. 4).

### (D) In mice infected with T. gondii sheep isolate

**Fig. 5:**
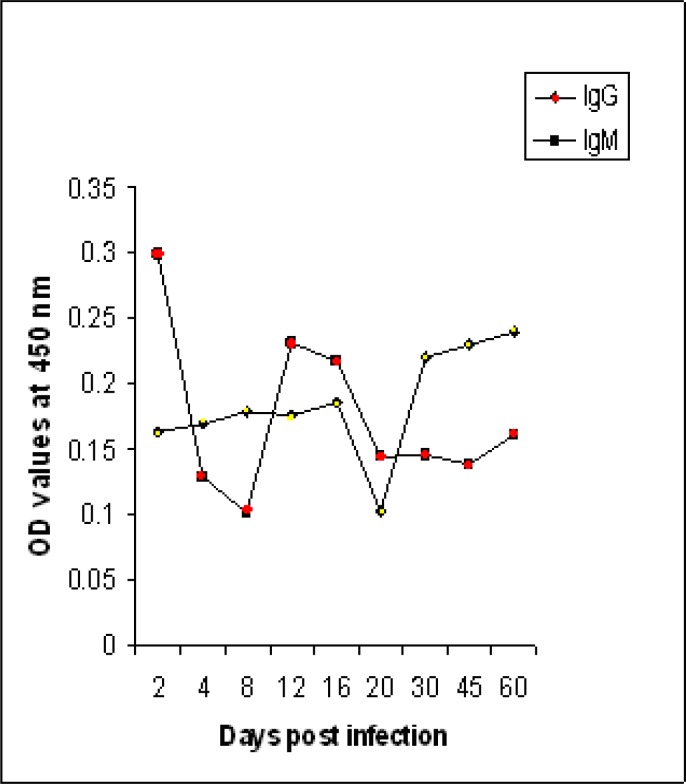
IgM and IgG antibodies levels to fraction 116 kDa of *T. gondii* RH strain in sera of mice experimentally infected with sheep isolate of *Toxoplasma gondii*

At early stage of infection, 2 days post infection, the fraction detected high level of IgM (0.299). Although the level decreased 4 and 8 days post infection, it was detectable until the end of the experiment. The profile of IgG was different than IgM but it was detected at early stage of infection at low level (0.163). It began to increase 30 days post infection and reached its peak (0.24) 60 days after infection (Fig. 5).

## Discussion

Crude *T. gondii* tachyzoites antigens represents as a raw material used to prepare fractions to be employed in different serologic tests for measurement of IgG and IgM antibodies in toxoplasmosis. So far, the actual antigenic molecules of the parasite involved in the interaction with *T. gondii* antibodies needs further investigations, where antigens of *Toxoplasma* are complex and diverse. The interactions between *T. gondii* legends and receptors on its target cells were depend on the wide range of hosts susceptible to *T. gondii* (26).

Searching for the appropriate diagnostic antigen for toxoplasmosis, an affinity partially purified antigen from *T. gondii* RH strain was adopted in the current study. CNBr-Sepharose 4B affinity column chromatography was previously utilized in the purification of *Toxoplasma* antigen by Ahn et al. (9), who coupled Sepharose 4B to IgG antibodies from rabbit immunized with crude antigen of *T. gondii* (RH strain). Actually, the profile of eluted fraction either structurally or immunologically depends mainly on the utilized antibodies and the tachyzoites of isolate used in the preparation of crude antigen. This was perfectly explained by results observed in the study of Abdel-Rahman et al. (12), who coupled horse sera with CNBr-Sepharose 4B to isolate four immunogenic bands of 136, 74, 44 and 31 kDa from *T. gondii* isolated from sheep. The isolated fraction was different from that isolated by affinity column chromatography in which Sepharose was coupled with horse serum to isolate fraction from crude antigen of horse *T. gondii* tachyzoites (5). Unexpectedly, the isolated fraction of sheep origin showed lower diagnostic potentials than its unbound fraction, the unidentified fraction by horse sera (12). Those results were supporting the postulation of the role of both antibodies and crude antigens in selection of particular fraction.

SDS-PAGE was utilized to differentiate between different eluted fractions from affinity columns chromatography. In the current study, the isolated fraction by infected mice sera consisted of a single band of approximately 116 kDa as proofed by SDS-PAGE. While SDS-PAGE analysis of the fraction recognized by IgG antibodies of immunized rabbit revealed four protein bands; 63, 53, 45 and 35 kDa (9). Six bands were isolated from *T. gondii* antigen of sheep origin with molecular weight ranged from 23 to 154.5 kDa identified by naturally infected horse sera (12). Another six bands with different molecular weights 66.2, 71, 83.2, 115.1, 130.5 and 155.8 kDa were isolated from *T. gondii* antigen of horse origin identified by infected horse sera (5). These observations further support the role of antibody and antigen in selection of a particular fraction in affinity column chromatography.

The diagnostic profile of 116 kDa isolated fraction from *T. gondii* RH strain was assessed by indirect ELISA in mice experimentally infected with RH and two local *T. gondii* isolates. The assay proofed its diagnostic potentials of toxoplasmosis due RH strain and the two isolates. IgM and IgG levels were higher in mice experimentally infected with RH strain than levels raised against other isolates except IgM level in sheep isolate. This observation has two faces; first, it proofed potency of the RH fraction in detecting mice toxoplasmosis due to other strains and the second concerned with strain dependent responses. Strain-dependence response has been observed for a range of phenotypes including blockade of host apoptosis, evasion of p47 GTPase-mediated killing and production of IL-12 (26).

For further explanation, strain-dependence response has been previously observed in mice (27–29). The reasons behind this observation reside in host-parasite relationship that was previously reported (30–35). Strain dependent humoral responses were observed in the present study represented in higher responses of IgM and IgG to RH fraction in mice infected with RH strain than their levels due to other strains. Recently, different responses (pathogenesis) in different mice strains infected with different doses of *T. gondii* strains of different genotypes were observed by Dubey et al., (36) supporting the concept of the current study.

Collectively, the present study introduces a 116 kDa affinity isolated fraction from *T. gondii* RH strain that successfully utilized to detect IgG and IgM responses in mice experimentally infected with RH strain of the parasite. Despite the humoral response in mice is strain dependent, the RH fraction detected IgG and IgM in mice infected with other strains supporting the suggestion declared by Smith and Frenkel (37), that only one immune-type of *Toxoplasma* is prevalent perhaps all over the world.

## Conclusion

The current research introduces an affinity isolated fraction from RH strain of *Toxoplasma gondii* consisted of a single band of 116kDa. The fraction proved potent diagnostic potentials of acute and chronic mice toxoplasmosis and it succeeded in diagnosis infection due to RH and two local isolates.
